# Case report: A 50 year‐old Down syndrome man exhibiting no Alzheimer’s disease biomarker profile nor cognitive impairment. Experience from the Argentine Initiative for the Study of Down syndrome and Alzheimer’s

**DOI:** 10.1002/alz.086949

**Published:** 2025-01-09

**Authors:** Giulia Solange Clas, Lucia Pertierra, Belén Helou, Fernanda Tapajoz, Nahuel Magrath Guimet, Ricardo Allegri, Mariela Marazita, Leonardo Romorini, Ezequiel Ignacio Surace

**Affiliations:** ^1^ Fleni, Buenos Aires, Buenos Aires Argentina; ^2^ Fleni, Buenos Aires Argentina; ^3^ FLENI, Escobar, Buenos Aires Argentina

## Abstract

**Background:**

Down Syndrome (DS) is the most common genetic form of intellectual disability. In recent years, there has been a significant increase in the life expectancy of individuals with DS, currently reaching the age of 60 or over. However, it has been observed that as of age 40, these individuals experience higher risk of developing dementia, and almost all of them exhibit histopathological characteristics of Alzheimer's disease (AD) in their brains.

As part of the first Latin American initiative for the study of Alzheimer’s disease in adults with Down syndrome (IASDA), we recruited a 50‐year‐old participant identified as SD8, who did not exhibit beta‐amyloid brain deposition and showed no signs of cognitive impairment.

**Method:**

The absence of beta‐amyloid brain deposition was determined by positron‐emission tomography (PET) using an amyloid‐binding compound (PiB) and quantification of cerebrospinal fluid biomarkers. To explore the possibility that lack of Aβ deposition could be due to an interstitial deletion of the *APP* gene, we performed Array‐CGH and MLPA assays. Additionally, an exome analysis was conducted to assess the presence of Alzheimer's protective gene variants. Induced pluripotent stem cells (iPSCs) were generated from SD8 peripheral blood mononuclear cells and subsequently differentiated into neurons. This approach will enable a more in‐depth study to gain further insights into this case.

**Result:**

These investigations revealed that SD8 possessed three copies of the APP gene, thus eliminating the possibility of an interstitial deletion. However, the exome analysis yielded no significant findings.

**Conclusion:**

In summary, in this study we present the strategies employed to delve into the possible mechanisms of SD8 being an AD “escapee” and outline our planned steps for further investigation.

